# Stem Cell-Derived, microRNA-Carrying Extracellular Vesicles: A Novel Approach to Interfering with Mesangial Cell Collagen Production in a Hyperglycaemic Setting

**DOI:** 10.1371/journal.pone.0162417

**Published:** 2016-09-09

**Authors:** Sara Gallo, Maddalena Gili, Giusy Lombardo, Alberto Rossetti, Arturo Rosso, Patrizia Dentelli, Gabriele Togliatto, Maria Chiara Deregibus, Daniela Taverna, Giovanni Camussi, Maria Felice Brizzi

**Affiliations:** 1 Department of Medical Sciences, University of Turin, Turin, Italy; 2 Department of Molecular Biotechnology and Health Sciences, University of Turin, Turin, Italy; Universita degli Studi di Palermo, ITALY

## Abstract

Extracellular vesicles (EVs) that are derived from stem cells are proving to be promising therapeutic options. We herein investigate the therapeutic potential of EVs that have been derived from different stem cell sources, bone-marrow (MSC) and human liver (HLSC), on mesangial cells (MCs) exposed to hyperglycaemia. By expressing a dominant negative STAT5 construct (ΔNSTAT5) in HG-cultured MCs, we have demonstrated that miR-21 expression is under the control of STAT5, which translates into Transforming Growth Factor beta (TGFβ) expression and collagen production. A number of approaches have been used to show that both MSC- and HLSC-derived EVs protect MCs from HG-induced damage via the transfer of miR-222. This resulted in STAT5 down-regulation and a decrease in miR-21 content, TGFβ expression and matrix protein synthesis within MCs. Moreover, we demonstrate that changes in the balance between miR-21 and miR-100 in the recipient cell, which are caused by the transfer of EV cargo, further contribute to providing beneficial effects. Interestingly, these effects were only detected in HG-cultured cells. Finally, it was found that HG reduced the expression of the nuclear encoded mitochondrial electron transport chain (ETC) components, CoxIV. It is worth noting that EV administration can rescue CoxIV expression in HG-cultured MCs. These results thus demonstrate that both MSC- and HLSC-derived EVs transfer the machinery needed to preserve MCs from HG-mediated damage. This occurs via the horizontal transfer of functional miR-222 which directly interferes with damaging cues. Moreover, our data indicate that the release of EV cargo into recipient cells provides additional therapeutic advantages against harmful mitochondrial signals.

## Introduction

Diabetes is the main driver of chronic kidney disease (CKD) in the Western world and accounts for about 50% of new cases. Almost 40% of diabetes sufferers develop diabetic nephropathy (DN), which has thus become the leading cause of end stage renal disease (ESRD) in urbanized countries [1]. Patients with CKD are not only at an increased risk of end-stage renal disease, but also cardiovascular disease and death [2,3]. Novel targets for improved DN management urgently need to be identified as ESRD can manifest despite strict glycaemic control and the application of various therapeutic approaches [4]. Key, early stage DN pathological features include podocyte damage/loss and mesangial cell (MC) hypertrophy [5]. The subsequent expansion of the myofibroblast progenitor population inside kidney stroma and increased extracellular matrix (ECM) protein synthesis lead to glomerular basement membrane thickening and tubulo-interstitial fibrosis [5,6]. A number of miRs have been reported to contribute to fibrotic processes in various pathological contexts, including DN [7]. In fact, miR-21 has gained particular interest in the field of MC expansion [8,9]. Osipova et al. [10] have recently reported increased concentrations of miR-21 in the urine of diabetic patients. Various miR-21 targets have been reported to further collagen production and fibrosis [8,9,11–13], while PTEN up-regulation, which results in the activation of the Akt-mTOR pathway, seems to be the principle contributor to this process [9,14]. As a matter of fact, it has been found that interfering with miR-21 reverses histological kidney abnormalities in a preclinical model of DN [8,12]. miR genes, like other genes, can be regulated by transcription factors [15]. In this regard, miR-21 has been described as a STAT5 target gene in Jurkat cells [16], as well as in mammary cells, in response to prolactin [17]. On the other hand, STAT5 itself can be controlled by miRs, including miR-222 [18,19], and miR-223 [20], which suggests that the overall scenario is extremely complex.

Clinical and experimental nephrologists are working in different fields to improve CKD outcomes. In particular, essential research is underway, using *in vivo* and *in vitro* models, which is aimed at defining the molecular basis for the principal pathways involved in CKD progression to ESRD and finding new therapeutic approaches to inhibiting renal fibrosis. Mesenchymal stem cells (MSCs) of different origin are currently being extensively studied in the regenerative medicine field [21]. Although MSCs were originally thought to home in on and engraft injured tissues, where they would differentiate and replace damaged cells, the positive effects of MSC transplantation have recently been proven to result from their ability to release trophic mediators [21]. Several studies have focused on extracellular RNA (exRNA) transporters and have indicated that they may be present in biological fluids in the form of vesicles, including exosomes and microvesicles [22,23]. The inclusive term “extracellular vesicles” (EVs) has been suggested [22,23] for these vesicles as they share overlapping features and biological activity despite having distinct biogenesis. EVs’ role as a well-preserved evolutionary mechanism of cell-to-cell communication has recently attracted increased attention [24]. In particular, it has been suggested that stem cell-derived EVs may mimic the effect of their parent cells via the horizontal transfer of functional RNAs, miRs, lipids and proteins in regenerative medicine [22–26]. A comparative analysis of EV cargo and stem cell sources indicates that the specific, rather than random, compartmentalization of different RNA species occurs [27]. The interaction between Alix, a multifunctional protein of the endosomal sorting complex required for transport, and Ago2 has recently been studied and its involvement in driving miRNAs within EVs has been suggested [28].

Although the functions of stem cell-derived EVs have been studied in various pathological settings, the mechanics of how EVs protect cells from damaging cues have only partially been investigated.

In the present study, we analyse the effects of EVs that were released from different stem cell sources on HG-induced collagen production and mitochondria damage, while paying particular attention to their effects on STAT5A, miR-21, miR-222, miR-100 and TGFβ expression.

## Materials and Methods

Reagents and antibodies are reported in S1 Table.

### Cell cultures

Human mesangial cells (MCs), characterized as previously described [29], were maintained in DMEM+10% FBS. MCs were either cultured at low (1 g/l, 5mmol/l, LG) or high D-glucose dose (4.5 g/l, 25mmol/l, HG) for 48h for the experiments. MCs in LG or HG DMEM were also cultured for an additional 18h without FBS, either in the presence or the absence of either bone-marrow-derived mesenchymal stem cell (MSC) (purchased from LONZA, Euroclone) [25,27] or human liver stem cell (HLSC)-derived EVs, isolated from adult human hepatocytes (purchased from LONZA) as previously described [30]. The same number of EVs was used to stimulate MCs (7000 EVs/ target cell) [31]. Endothelial cells (ECs) cultured for 48h with ox-LDL (10 ng/ml) were used as a positive control for the senescence assay. MCs treated for 48h with TGFβ (10 ng/ml) were used as positive control for proliferation assay and for the expression of collagen. Human fibroblasts cultured 24 hours in HG medium were used as positive control for TGFβ production.

All experiments were performed in accordance with the European Guidelines and policies and approved by the Ethical Committee of the University of Turin.

### Isolation and quantification of MSC- and HLSC-derived EVs

MSCs and HLSCs were cultured in EndoGRO Medium, without FBS, for 24 h in order to collect EVs from supernatants. After being centrifuged at 3000 *g* for 30 min to remove debris, cell-free supernatants were submitted to differential ultracentrifugation at 10 000 and 100 000 *g* (Beckman Coulter Optima L-90K ultracentrifuge; Beckman Coulter, Fullerton, CA, USA) for 3h at 4°C. EVs were either used fresh or stored (-80°C) after re-suspension in DMEM supplied with 1% DMSO [26]. Frozen EVs were washed and pelleted by 100k g ultracentrifugation to remove DMSO before experiments. No difference in biological activity was observed between fresh and stored EVs. EV protein content was quantified using the Bradford method (Bio-Rad, Hercules, CA, USA). Possible contamination was tested for using a Limulus amebocyte assay (concentration <0.1 ng/ml) (Charles River Laboratories, Inc., Wilmington, MA, USA). EV size distribution analyses were performed using a NanoSight LM10 (NanoSight Ltd, Minton Park UK). The particles in the samples were illuminated using a laser light source and the scattered light was captured by camera and analysed using Nanoparticle Tracking Analysis (NTA). NTA automatically tracked and sized particles according to Brownian motion and the diffusion coefficient (Dt). EV characterization has been performed by Collino et al [27].

### Cell proliferation

Proliferative activity in both LG and HG conditions, with or without EVs, was assayed via direct cell count by 3 individual operators in triplicate, as previously described (number±SEM of cells per field, 10X magnification) [32].

### Western blot analysis

MCs were lysed and protein concentrations obtained as previously described [33]. 50 μg proteins were subjected to SDS-PAGE, transferred into nitrocellulose membranes and processed as previously described [33]. Densitometric analysis was used to calculate the differences in the fold induction of protein levels which were normalized to β-actin. Values are reported as relative amounts. To evaluate CoxIV content, MCs were lysed in RIPA buffer (Sigma-Aldrich) and subjected to western blot. Cell supernatants from LG- or HG-cultured MCs were collected, centrifuged to remove cell debris and analysed by western blot for collagen type IV content.

### RNA isolation and quantitative real-time PCR (qRT-PCR)

Total RNA was isolated from MCs using the TRIzol reagent (Invitrogen) according to manufacturer’s instructions. RNA was quantified spectrophotometrically (Nanodrop ND-1000, Wilmington, DE, USA). RNA from cells was then reverse-transcribed either using a TaqMan microRNA RT kit, that is specific for miR-222 and miR-223, or a Syber Green microRNA RT Kit specific for miR-21 and miR-100. Thus RNA was subjected to qRT-PCR using a TaqMan/Syber microRNA assay kit and the ABI PRISM 7700 sequence detection system (Applied Biosystems, Foster City, CA, USA). miR expression was normalized to the small nuclear RNA, RNU6B.

### Gain- and loss-of-function experiments

Gain- and loss-of-function experiments were performed in MCs cultured in LG or HG that had either been transfected with the pre-miR negative control or the pre-miR-222, pre-miR-223 or pre-miR-100 precursor oligonucleotides (Applied Biosystem), according to manufacturer’s instructions. LG-cultured MCs were also transfected with either a pre-miR control or pre-miR-21, whereas HG-cultured MCs were transfected with anti-miR negative control or anti-miR-21 oligonucleotides. Cells were then either processed to give the cell extracts for Western blot analyses or total RNA isolation to evaluate miR expression.

### Transfer of miRs from EVs to MCs

To analyse miR-222 transfer from EVs to MCs, miR transfer experiments were conducted as previously described by Yuan [34]. 5×10^5^ cells/well of MCs were incubated with a transcriptional inhibitor, α-amanitin [31], either in the absence or in the presence of EVs that had either been pre-treated with RNAse or not. Total RNA from MCs, was subjected to qRT-PCR for miR expression. As an indirect measure of miR transfer, we determined the difference in Ct values between α-amanitin treated cells in the absence and in the presence of EVs that had either been pre-treated with RNAse or not; a positive value indicated transfer of miR into target cells. If no signal was detected, a Ct value of 40 was assigned to the sample.

### Transfection of dominant negative (ΔN) STAT5 construct

In selected experiments, HG-cultured MCs were either transiently transfected with an empty vector or with the ΔNSTAT5 construct for 48h [35]. Cells were then processed for Western blot analysis or RNA isolation.

### Luciferase miRNA Target Reporter Assay

The luciferase reporter assay was performed using a construct generated by sub cloning PCR products amplified from 3’UTR full length of STAT5A mRNA in the Sac I restriction site of the luciferase reporter vector pGL3 (Promega, Madison, WI, USA). PCR products were obtained using the following primers: STAT5A: sense, 5’AAGAGCTCATGTTTGAATCCCACGCT3’; antisense, 5’TTGAGCTCACACAAATGTGTGGTCTT3’. The pGL3, pGL3-3’UTR STAT5A luciferase reporter vectors were transiently co-transfected in MCs, treated as indicated, at 10:1 molar ratio with the pRL vector, coding for the Renilla luciferase, used as internal control of the luciferase assay [18]. Luciferase activities, using the pGL3 reporter vectors, above described, were also evaluated in MCs transfected with pre-miR-222.

### Senescence assay

Senescence was evaluated by measuring the acidic β-galactosidase activity of MCs that had been differently cultured, as previously described [36].

### Statistical analysis

All data are presented as mean or percentage±SEM. The D'Agostino—Pearson test was used to test normality. Data were analysed using the Student's *t*-tests for two-group comparison and using a one-way analysis of variance, followed by Tukey's multiple comparison test for 3 groups. Three experiments performed in triplicate were the minimum sample size ensuring 90% statistical power between experimental groups, with a probability level of 0.05, two-tailed hypothesis. The cut-off for statistical significance was set at *p*<0.05. All statistical analyses were carried out using GraphPad Prism version 5.04 (GraphPad Software, Inc., La Jolla, CA, USA).

## Results

### High glucose stimulation induces mesangial cell (MC) proliferation, collagen type IV production and miR-21 expression

**Fig 1:**
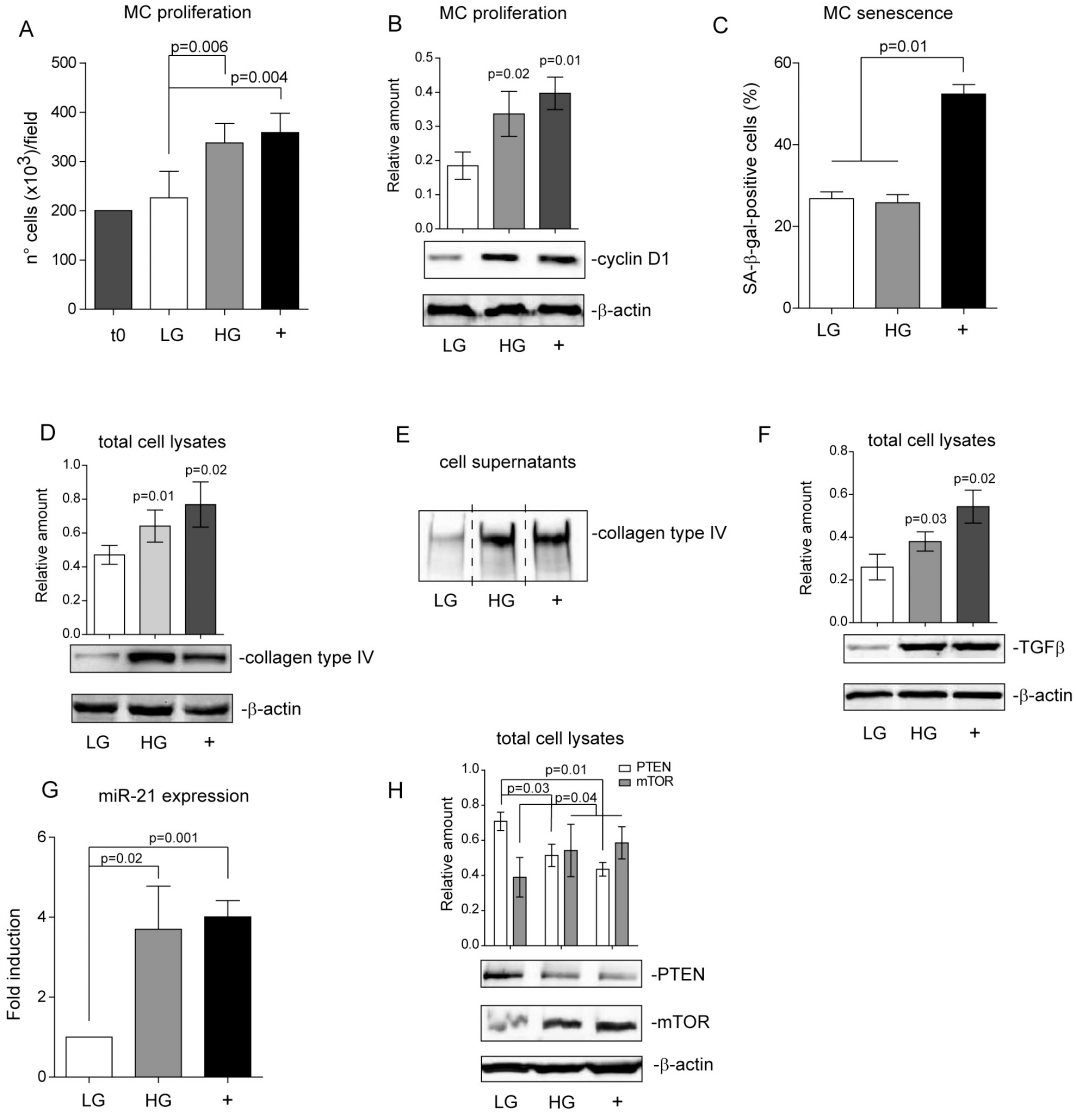
Mesangial cell (MC) proliferation and collagen type IV production upon short term high glucose (HG) treatment. **(A)** A cell proliferation assay was performed in MCs treated for 48h as indicated and reported as number of cells per field (20X magnification). Data are expressed as mean±SEM (n = 6) (*p = 0*.*006*, HG-treated MCs vs LG-treated MCs; *p = 0*.*004*, TGFβ-treated MCs (+; 10 ng/ml) vs LG-treated MCs). **(B)** Cell extracts from MCs treated with either LG, HG or TGFβ (+) were analysed for cyclin D1 content, normalized to β-actin content (*p = 0*.*02*, HG-treated MCs vs LG-treated MCs; *p = 0*.*01*, TGFβ-treated MCs (+) vs LG-treated MCs, n = 4). **(C)** Senescence was evaluated on MCs cultured for 48h as indicated and expressed as the percentage of SA-β-gal-positive cells. Endothelial cells treated with ox-LDL were used as a positive control (*p = 0*.*01*, LG- and HG-treated MCs vs positive control (+), n = 5). **(D)** Cell extracts from MCs treated as above were analysed for collagen type IV content, normalized to β-actin (*p = 0*.*01*, HG-treated MCs vs LG-treated MCs; *p = 0*.*02*, TGFβ-treated MCs (+) vs LG-treated MCs, n = 6). **(E)** Cell supernatants recovered from MCs treated as above were analysed by western blot for collagen type IV content (*p = 0*.*01*, HG and TGFβ (+) vs LG treated MCs, n = 5). **(F)** TGFβ content was analysed by western blot on MCs treated as above. Fibroblasts treated with HG were used as a positive control. Data were normalized to β-actin content (*p = 0*.*03*, HG vs LG treated MCs; *p = 0*.*02*, HG-treated fibroblasts vs LG-treated MCs, n = 5). **(G)** miR‐21 expression was evaluated by qRT‐PCR on MCs that had been treated as above. Data normalized to RNU6B are representative of six experiments performed in triplicate (*p = 0*.*02*, HG vs LG treated MCs; *p = 0,001* TGFβ (+) vs LG-treated MCs). **(H)** Cell extracts from treated MCs were analysed for PTEN and mTOR content and normalized to β-actin content (*p = 0*.*03*, HG vs LG and *p = 0*.*01* TGFβ vs LG treated MCs for PTEN; *p = 0*.*04*, HG and TGFβ vs LG treated MCs for mTOR, n = 4).

MCs were cultured in high glucose medium for 48 hours in order to mimic acute hyperglycaemia-mediated mesangial cell damage. Proliferation and senescence were then analysed. We demonstrate that HG significantly increases MC proliferation (Fig 1A), as shown by cell number and cyclin D1 content (Fig 1B). No changes in the number of senescent MCs were detected (Fig 1C). Collagen production was evaluated in MCs challenged with HG as collagen production is a hallmark of glomerular damage [5,6]. Western blot analysis on both total cell lysates and supernatants showed that collagen type IV production significantly increased compared to low glucose treated cells (Fig 1D and 1E). The increase in collagen production indicates that MCs shift to a fibrotic secretive phenotype. TGFβ already barely produced by MCs, was further increased by HG treatment (Fig 1F). miR-21, well-known for being involved in diabetic nephropathy, is also known to induce mesangial cell matrix expansion [8,11]. In fact, we found that HG treatment induces miR-21 expression (Fig 1G). Its role in regulating the PTEN-mTOR signalling pathway has been reported in numerous cell types [8,9,11,14] leading us to investigate the involvement of this signalling pathway. As reported in Fig 1H, we found low level of PTEN and high level of mTOR even in HG treated MCs.

### EVs released from MSCs and HLSCs inhibit collagen production and miR-21 expression in MCs

**Fig 2:**
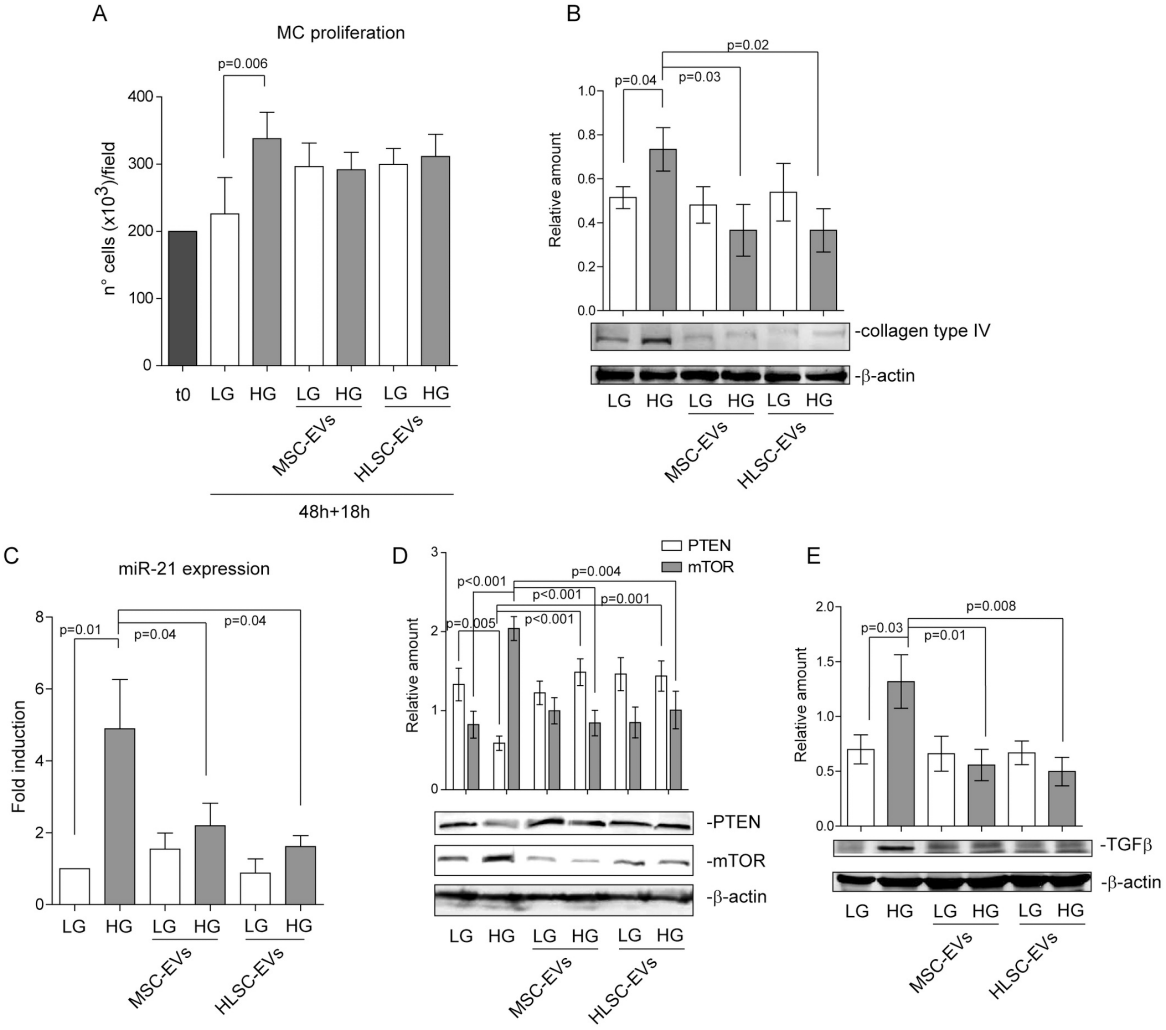
EVs recovered from MSCs and HLSCs interfere with collagen production and miR-21 expression in MCs. **(A)** Cell proliferation was assayed in MCs that had been cultured for 48h in either LG or HG conditions, then starved and either treated with the indicated EVs for 18h or left untreated. Data are reported as number of cells per field (20X magnification) and expressed as mean±SEM (n = 5) (*p = 0*.*006*, HG vs LG treated MCs). **(B)** Cell extracts from MCs treated as in A were analysed for collagen type IV content and normalized to β-actin content (n = 5) (*p = 0*.*04*, HG vs LG treated MCs; *p = 0*.*03*, HG-treated MCs+MSC-EVs vs HG-treated MCs; *p = 0*.*02*, HG-treated MCs + HLSC-EVs vs HG-treated MCs). **(C)** miR‐21 expression was evaluated by qRT‐PCR on 48h LG- or HG-cultured MCs, either untreated or treated with MSC-EVs or HLSC-EVs for 18h. Data normalized to RNU6B are representative of six experiments performed in triplicate (n = 6) (*p = 0*.*01*, HG vs LG treated MCs; *p = 0*.*04*, HG-treated MCs+MSC-EVs vs HG-treated MCs; *p = 0*.*04*, HG-treated MCs+HLSC-EVs vs HG-treated MCs). **(D)** Cell extracts from MCs treated as above were analysed for PTEN and mTOR content and normalized to β-actin (*p = 0*.*005*, HG vs LG treated MCs; *p<0*.*001*, HG-treated MCs+MSC-EVs vs HG-treated MCs; *p = 0*.*001*, HG-treated MCs+HLSC-EVs vs HG-treated MCs for PTEN; *p<0*.*001*, HG vs LG treated MCs; *p<0*.*001*, HG-treated MCs+MSC-EVs vs HG-treated MCs; *p = 0*.*004*, HG-treated MCs+HLSC-EVs vs HG-treated MCs for mTOR, n = 6). **(E)** Cell extracts from MCs treated as indicated were analysed for TGFβ content, normalized to β-actin (n = 5) (*p = 0*.*03*, HG vs LG treated MCs; *p = 0*.*01*, HG-treated MCs+MSC-EVs vs HG-treated MCs; *p = 0*.*008*, HG-treated MCs+HLSC-EVs vs HG-treated MCs).

The effect of EVs recovered from MSCs and HLSCs on MC proliferation and collagen production in LG- and HG-cultured MCs was evaluated. EVs did not interfere with MC proliferation as shown in Fig 2A. However, EVs recovered from MSCs and HLSCs significantly reduced collagen production in MCs (Fig 2B). Furthermore, down-regulated miR-21 (Fig 2C), mTOR (Fig 2D) and TGFβ expression were detected in MCs (Fig 2E). Consistently, up-regulation of PTEN was detected (Fig 2D).

### MSC- and HLSC-derived EVs regulate STAT5A expression in MCs subjected to HG

**Fig 3:**
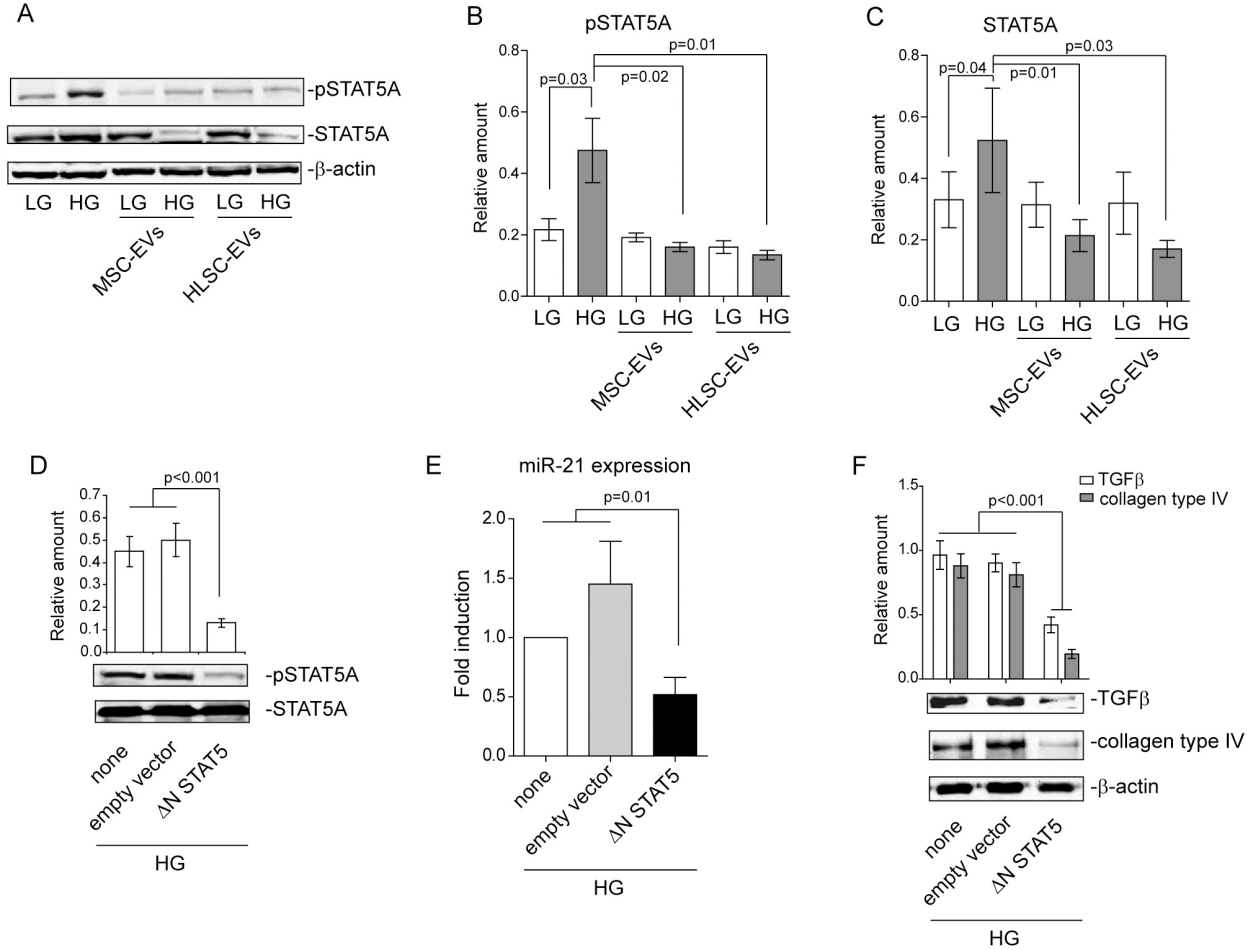
MSC- and HLSC-derived EVs drive STAT5A expression in MCs. **(A-C)** Cell extracts from 48h LG- or HG-cultured MCs treated with either MSC-EVs or HLSC-EVs for 18h were analysed for pSTAT5A and STAT5A content, normalized to β-actin (n = 4) (*p = 0*.*03*, HG vs LG treated MCs; *p = 0*.*02*, HG-treated MCs+MSC-EVs vs HG-treated MCs; *p = 0*.*01*, HG-treated MCs+HLSC-EVs vs HG-treated MCs for pSTAT5A in **B**. *p = 0*.*04*, HG vs LG treated MCs; *p = 0*.*01*, HG-treated MCs+MSC-EVs vs HG-treated MCs; *p = 0*.*03*, HG-treated MCs+HLSC-EVs vs HG-treated MCs for STAT5A in **C**). **(D)** Cell extracts from MCs cultured for 48h with HG and either transfected with ΔNSTAT5 construct, with the empty vector (48h) or not at all were subjected to SDS-PAGE to evaluate pSTAT5A content, normalized to STAT5A content (n = 4) (*p<0*.*001*, ΔNSTAT5 vs none and empty vector). **(E)** miR‐21 expression was evaluated by qRT‐PCR on MCs treated as in D. Data normalized to RNU6B are representative of six experiments performed in triplicate (n = 6) (*p = 0*.*01*, ΔNSTAT5 vs none and empty vector). **(F)** Cell extracts from HG-cultured MCs, either transfected with ΔNSTAT5 construct, with the empty vector for 48h or not at all, were analysed for TGFβ and collagen type IV content, normalized to β-actin (n = 5) (*p<0*.*001*, ΔNSTAT5 vs none and empty vector for TGFβ and collagen type IV).

We have previously shown that delayed (48 hours) HG treatment leads to STAT5 activation in MCs [33]. It has been shown that miR-21 expression falls under the control of STAT5 in response to prolactin [17]. The observation that EVs derived from MSCs and HLSCs affect miR-21 expression has led us to investigate whether STAT5 may be involved in its regulation. To this purpose, MCs treated with HG were analysed for STAT5A activation, either in the presence or absence of EVs. As shown in Fig 3A, STAT5A underwent activation in response to HG treatment, but this effect was inhibited by EV treatment (Fig 3B). The observation that reduced STAT5A expression was detected in these experimental conditions (Fig 3C) enforces the possibility that EV cargo regulates its expression. In order to investigate this possibility further, a ΔNSTAT5 construct was transfected into MCs (Fig 3D) and miR-21 expression was analysed. As reported in Fig 3E, the inhibition of STAT5A activation leads to miR-21 down-regulation in HG-cultured MCs. Moreover, collagen production was found to be almost completely suppressed when analysed in these experimental conditions (Fig 3F). Interestingly, we also found that the inhibition of the STAT5 signalling pathway prevents HG-mediated TGFβ expression (Fig 3F).

### EV miR cargo regulates STAT5A expression

**Fig 4:**
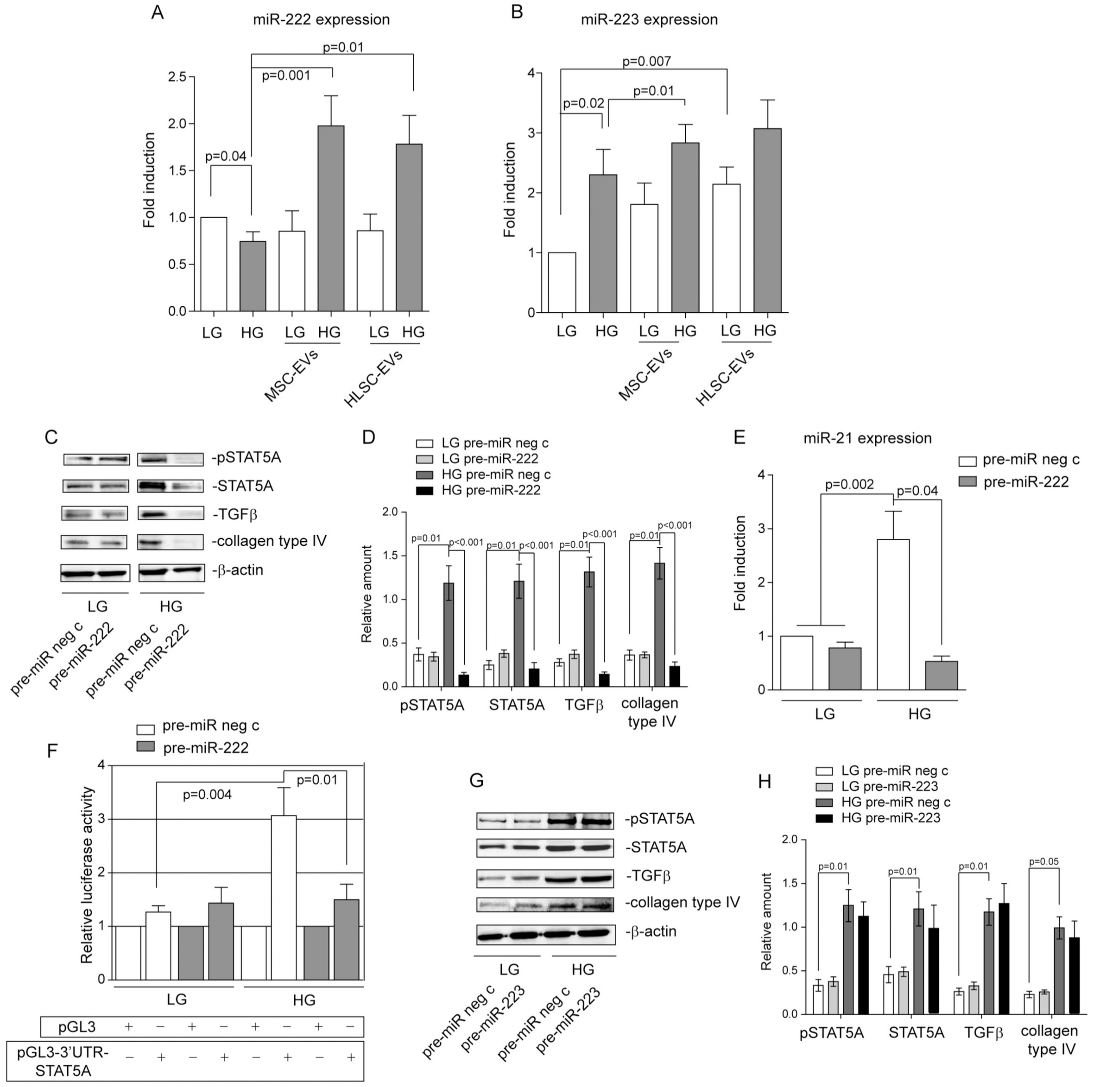
miR-222 EV cargo drives STAT5A expression in HG-cultured MCs. **(A-B)** miR‐222 (A) and miR-223 (B) expression was evaluated by qRT‐PCR on 48h LG- or HG-cultured MCs, that had either been treated with MSC-EVs or HLSC-EVs for 18h or otherwise left untreated. Data normalized to RNU6B are representative of six experiments performed in triplicate (n = 6) (*p = 0*.*04*, HG vs LG; *p = 0*.*001*, HG-treated MCs+MSC-EVs vs HG-treated MCs; *p = 0*.*01*, HG-treated MCs+HLSC-EVs vs HG-treated MCs in **A**: *p = 0*.*02*, HG vs LG; *p = 0*.*01*, HG-treated MCs+MSC-EVs vs HG-treated MCs; *p = 0*.*007*, LG-treated MCs+HLSC-EVs vs LG-treated MCs in **B**). **(C)** Cell extracts from MCs, transfected with either pre-miR neg control or pre-miR-222 and cultured in either a LG or HG medium were subjected to SDS-PAGE and analysed for pSTAT5A, STAT5A TGFβ and collagen type IV content, normalized to β-actin content. **(D)** Densitometric analysis was performed on western blots of MCs treated as above (n = 5) (LG pre-miR neg control vs HG pre-miR neg c, *p = 0*.*01* for pSTAT5A, STAT5A, TGFβ and collagen type IV content; HG pre-miR neg c vs HG pre-miR-222, *p<0*.*001* for pSTAT5A, STAT5A, TGFβ and collagen type IV). **(E)** miR‐21 expression was evaluated by qRT‐PCR on MCs, transfected with pre-miR neg c or pre-miR-222, cultured in either a LG or HG medium. Data were normalized to RNU6B (five experiments performed in triplicate, n = 5) (*p = 0*.*002*, LG pre—miR neg c and LG pre-miR-222 vs HG pre-miR neg c; *p = 0*.*04*, pre-miR-222 vs pre miR neg in HG). **(F)** MCs were either transfected with pGL3 empty vector or pGL3‐3′‐UTR-full length-STAT5A luciferase constructs, treated as indicated. Relative luciferase activity is reported as mean±SEM (n = 4) (*p = 0*.*004*, LG pre-miR neg c vs HG pre-miR neg c for pGL3-3’UTR-STAT5A; *p = 0*.*01*, HG pre-miR-222 vs HG pre miR neg c). **(G)** Cell extracts from LG- or HG-cultured MCs, transfected with pre-miR neg control or pre-miR-223, were subjected to SDS-PAGE and analysed for pSTAT5A, STAT5A, TGFβ and collagen type IV content, normalized to β-actin content. **(H)** Densitometric analysis was performed on western blots of MCs treated as above (n = 5) (LG pre-miR neg c vs HG pre-miR neg c, *p = 0*.*01* for pSTAT5A, STAT5A and TGFβ content; *p = 0*.*05*, for collagen type IV content).

MSC and HLSC EV miRnomics have been previously reported [27]. We evaluated whether EV-borne-miRs, and in particular miR-223 and miR-222, are relevant to regulating the signalling pathway that is activated by HG. It has been reported that both miR-223 and miR-222 post-transcriptionally regulate STAT5 [18–20], which led us to evaluate their expression in HG-cultured, EV treated MCs. Whereas miR-222 is down regulated upon HG treatment and increased upon EV treatment (Fig 4A), miR-223 increased upon both HG and EV treatment (Fig 4B). Gain-of function experiments were performed in MCs transfected with pre-miR-222 or pre-miR-223 and cultured in LG or HG conditions (S1A and S1B Fig), in order to evaluate which miR was able to regulate STAT5 expression. Interestingly, only miR-222 over-expression reduced STAT5A expression and activation, the consequent miR-21 level, TGFβ expression and collagen production in HG-treated MCs (Fig 4C–4H). The luciferase assay further supports the role of miR-222 in driving STAT5 post-transcriptional regulation (Fig 4F). More importantly, such events did not occur in LG-cultured MCs. The transfer of miR-222 into MCs by EVs was demonstrated by experiments performed in the presence of α-amanitin, using EVs that had either been pre-treated with RNAse or not at all (S1C Fig).

### EV-mediated miR-21 down-regulation can promote miR-100 post-transcriptional activity which contributes to the inhibition of collagen production

**Fig 5:**
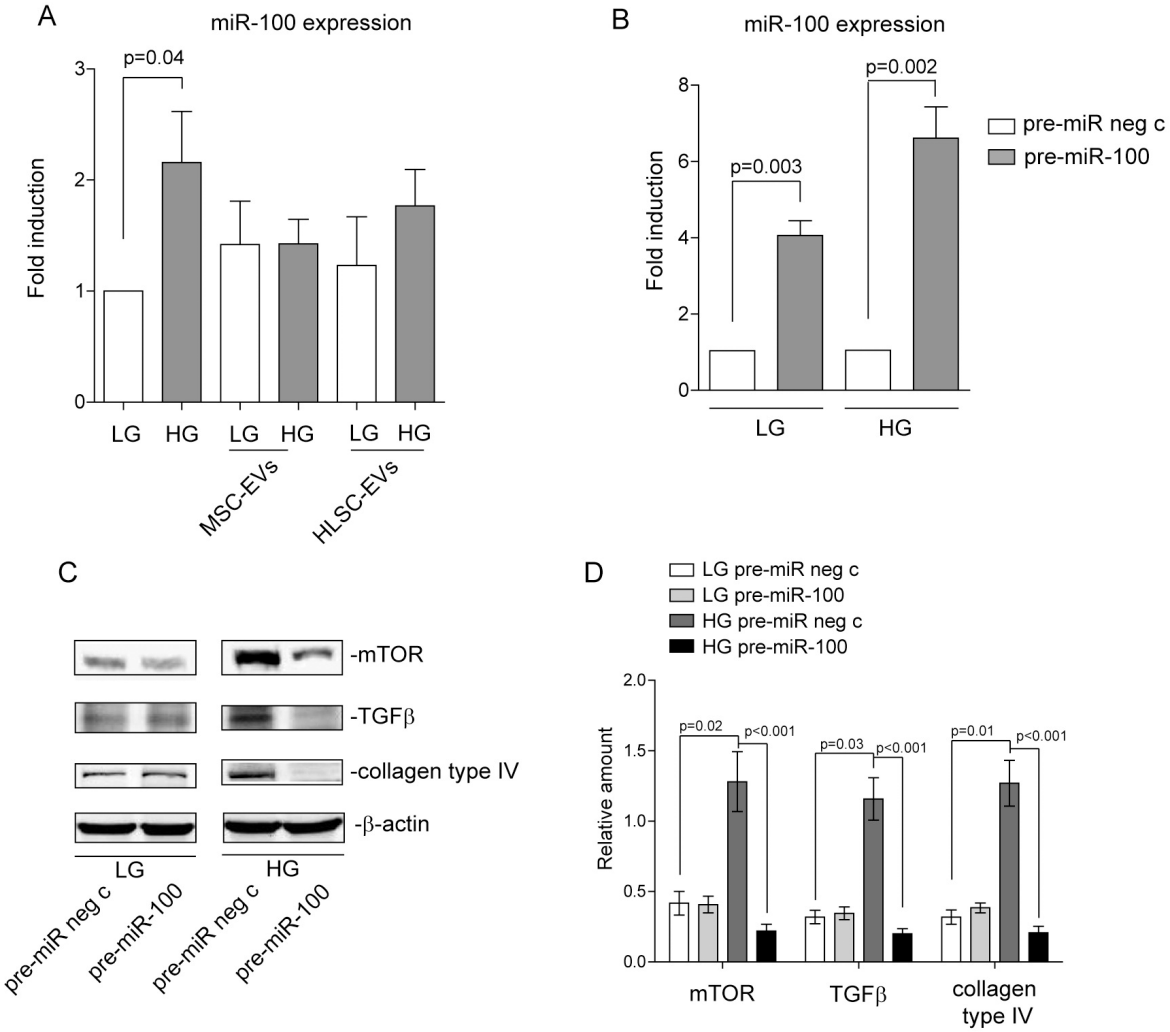
Over-expression of miR-100 in HG-treated MCs impairs mTOR and TGFβ expression and collagen production. **(A)** miR-100 expression was evaluated by qRT-PCR on 48h LG- and HG-cultured MCs, either alone or in combination with MSC-EVs or HLSC-EVs for 18h. Data normalized to RNU6B are representative of four different experiments performed in triplicate (n = 4). (*p = 0*.*04*, LG vs HG treated MCs). **(B)** Gain-of-function experiments were performed on MCs cultured in LG or HG medium for 48h, using pre-miR-100 oligonucleotides. Pre-miR negative control (neg c) oligonucleotides were used as control. miR-100 expression was evaluated by qRT-PCR and normalized to RNU6B (five experiment performed in triplicate, n = 5) (*p = 0*.*003*, pre-miR-100 vs pre miR neg in LG; *p = 0*.*002* pre-miR-100 vs pre miR neg in HG. **(C)** Cell extracts from MCs cultured in either a LG or HG medium and transfected with either pre-miR neg c or pre-miR-100 were subjected to SDS-PAGE and analysed for mTOR, TGFβ and collagen type IV content, normalized to β-actin. **(D)** Densitometric analysis was performed on western blots of MCs treated as above (n = 6) (LG pre-miR neg c vs HG pre-miR neg c, *p = 0*.*02* for mTOR, *p = 0*.*03* for TGFβ and *p = 0*.*01* for collagen type IV content; HG pre-miR neg c vs HG pre-miR-100, *p<0*.*001* for mTOR, TGFβ and collagen type IV content).

The dual findings that miR-100 is also carried by MSC- and HSC-derived EVs [27], and that miR-100 post-transcriptionally regulates mTOR [37], led us to investigate whether it can also contribute to EV-mediated effects. First, miR-100 expression was evaluated in MCs subjected to EV treatment. As shown in Fig 5A, while miR-100 MC content increased upon HG treatment, no changes in its content were detected after EV treatment. However, as the balance of intracellular miRs directs specific biological responses, we hypothesized that the decreased miR-21 intracellular content associated with EV treatment could favour miR-100 post-transcriptional activity. To validate this hypothesis, gain-of function experiments using pre-miR-100 were performed in HG-treated MCs (Fig 5B). Indeed, data reported in Fig 5C and 5D demonstrate that miR-100 can, when its expression is favoured with respect to miR-21, drive signals, mediated by mTOR down-regulation, which result in the inhibition of MC TGFβ expression and collagen production. The effect of miR-100 over-expression did not further increase after EVs treatment (data not shown). Again, miR-100 over-expression did not impact on LG-cultured MCs (Fig 5C and 5D). Collectively, these data indicate that the fine tuning of miR content in recipient cells during EV treatment, might also contribute to the healing properties delivered.

### EV treatment boosts mitocondrial CoxIV expression in HG treated cells

**Fig 6:**
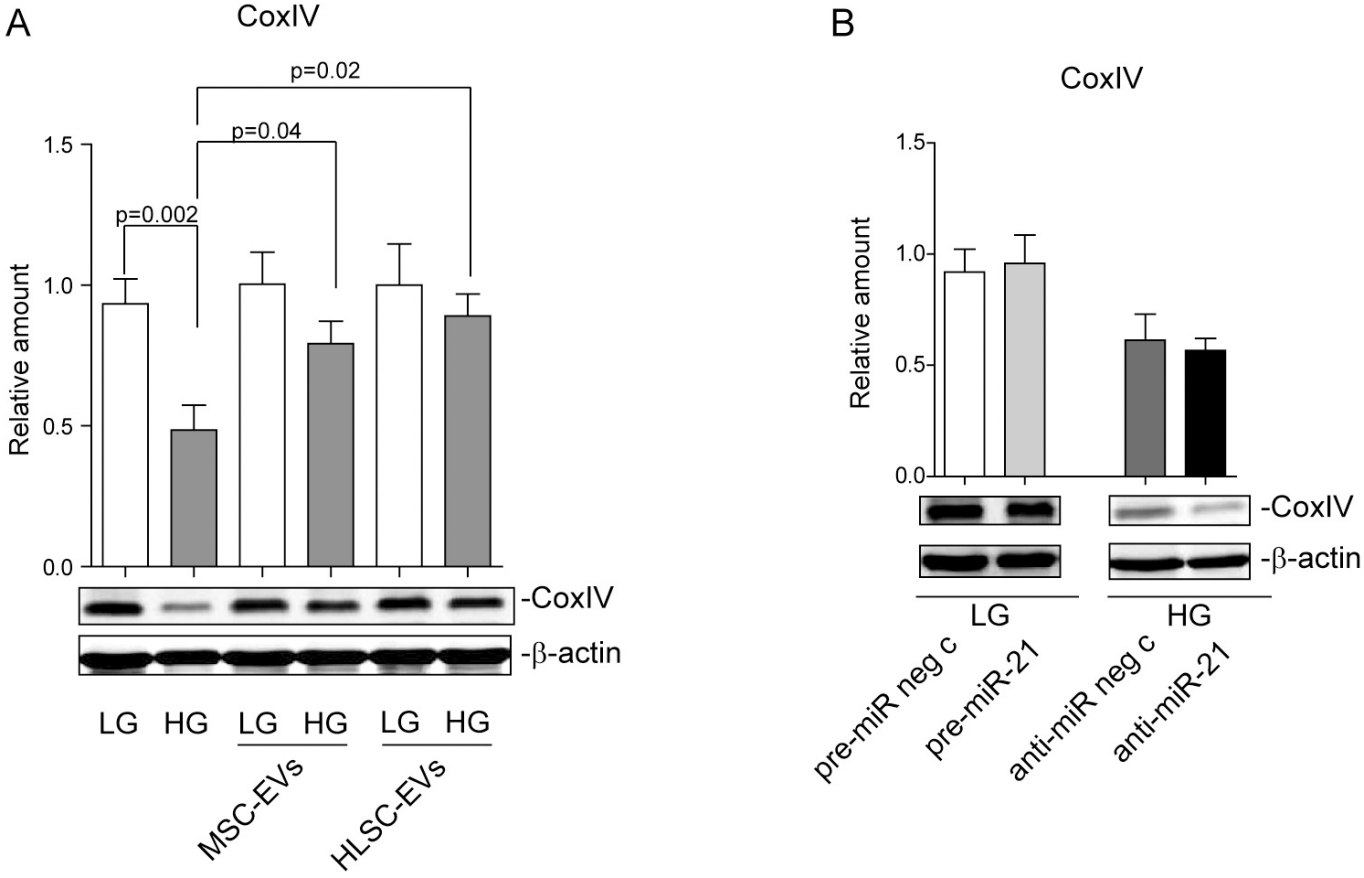
EV treatment boosts mitochondrial CoxIV expression in HG-treated cells. **(A)** LG- or HG-cultured MCs, that had either been treated with MSC-EVs, HLSC-EVs or not at all, were lysed in a RIPA buffer and analysed for CoxIV content, normalized to β-actin (n = 4) (*p = 0*.*002*, LG vs HG treated MCs; *p = 0*.*04*, HG-treated MCs+MSC-EVs vs HG-treated MCs; *p = 0*.*02*, HG-treated MCs+HLSC-EVs vs HG-treated MCs). **(B)** LG-cultured MCs that had been transfected with either pre-miR neg control or pre-miR-21 and HG-cultured MCs transfected with either anti-miR neg control or anti-miR-21 were lysed in a RIPA buffer and subjected to SDS-PAGE to evaluate CoxIV content, normalized to β-actin (n = 5).

The disturbance of mitochondrial homeostasis is a key event in HG-induced cellular damage [38]. The role of miR-21 in mitochondrial disorder has previously been reported [39]. We therefore decided to evaluate whether EVs were able to prevent the mitochondrial damage that is associated with HG-treatment. To this purpose, the expression of the CoxIV protein, one of the nuclear encoded mitochondrial electron transport chain (ETC) components, was evaluated in LG- or HG-cultured MCs that had either been treated with EVs or not at all. It was found that HG, unlike LG, reduced CoxIV expression. It is worth noting that EV administration can rescue CoxIV expression in HG-cultured MCs (Fig 6A). In order to investigate the role of miR-21 in regulating CoxIV expression, LG-cultured MCs were transfected with pre-miR-21 and analysed for CoxIV content. Interestingly, CoxIV expression was not affected by miR-21 over-expression (S1D Fig) in this experimental condition (Fig 6B). This suggests that miR-21 over-expression alone could be still balanced by a compensatory cell response [40]. As a matter of fact, a low CoxIV level was detected in HG-treated MCs that had been transfected with anti-miR-21 oligonecleotides (Fig 6B and S1E Fig).

## Discussion

The present study shows that EVs released from MSCs and HLSCs protect MCs from HG-induced collagen production. In particular, we found that EVs that transfer miR-222 regulate STAT5A expression that in turn controls miR-21 and TGFβ expression as well as matrix protein synthesis. Moreover, we provide evidence that EVs may also indirectly contribute to the inhibition of collagen production by driving changes in the balance between miR-21 and miR-100 in the recipient cell. Finally, we demonstrate that EVs can also save MCs from harmful mitochondrial signals. These results indicate that both MSC-and HLSC-derived EVs can transfer/activate the machinery needed to preserve MCs from HG-mediated damage.

The renal structural alterations in DN are characterized by an early high proliferation rate of both glomerular and tubular cells and the late accumulation of extracellular matrix proteins, such as collagen IV and fibronectin, which lead to progressive mesangial expansion [5,41].

Hyperglycaemia plays a crucial role in these processes and a number of mechanisms have been proposed [5,10,41]. MC proliferation is an early indicator of DN progression [5,10] and, as shown herein, HG induces MC proliferation. TGFβ has been recognized as the most relevant regulatory cytokine in the onset of MC collagen accumulation [42–44]. Indeed, 48 hour HG treatment led to TGFβ expression and collagen production. Likewise, miR-21, one of the most relevant miRs in the control of fibrogenic processes, was up-regulated upon HG treatment. miR-21 is over-expressed in a different model of fibrosis and diverse pathways drive its expression [6,9,11]. miR genes are regulated by transcriptional factors [15], just like other genes. The *miPPR21* gene is targeted by STAT3 [45], and STAT5 [17], which may initiate opposing biological actions; STAT3 inhibits, whereas STAT5 induces, *miPPR21* gene transcription [17,45]. In this regard, STAT5 binding to the *miPPR-21* gene has been shown to regulate miR-21 expression in Jurkat cells [16], and in mammary cells in response to prolactin [17]. In an experiment that uses data from Wang and collaborators [46], and which is consistent with our previous report [33], we have found that HG treatment induces delayed STAT5 activation, which occurs after 48 hours of treatment. This observation led us to investigate the impact of STAT5 on miR-21 expression. Indeed, MCs that are unable to activate STAT5A also failed to increase miR-21 content in response to HG. Moreover, we demonstrate that this effect translates into the inhibition of TGFβ expression and collagen production. These data are in line with the results published by Wang et al. [46], which show that a JAK2 blockade was only effective in inhibiting STAT5 activation 48 hours after HG treatment. Moreover, such delayed STAT5 activation kinetics could also explain our previous data [33]. Collectively, these results identify STAT5A as the transcriptional regulator of miR-21 which, in turn, impacts on TGFβ expression and collagen production in MCs subjected to HG. Moreover, since HG treatment recapitulates DM-induced MC damage, MCs might be a good cell model with which to assess the efficacy of novel therapeutic options. This would be of particular interest as so far as DN is becoming the leading cause of ESRD despite strict metabolic control and the application of various beneficial approaches and novel therapeutic options [4].

Regenerative medicine and, in particular, cell-based therapy have recently attracted special attention and various cell types have been proposed, including mesenchymal stem cells (MSCs) of different origin [21,47]. Their particular properties make MSC-derived extracellular vesicles (EVs) a promising new therapeutic option. Adult human stem cells, including MSCs and HLSCs, secrete EVs which contain specific subsets of functional miRs [27], with healing properties [24]. We herein demonstrate that EVs released from both MSCs and HLSCs can interfere with the signalling pathways activated in MCs by the hyperglycaemic microenvironment. In particular, we demonstrate that the transfer of EV cargo into MCs leads to reduced STAT5 expression/activation, the down-regulation of miR-21 and TGFβ expression which ultimately results in a reduction of MC collagen production. Furthermore, EV treatment was found to be associated with mTOR down-regulation. The direct EV-mediated delivery of miRs was discovered to affect the translation of target mRNA, which suggests that they may control cell biology [22,23,27,30]. An analysis of EV miR content in both cellular sources identified miR-222 and miR-223 [27]. It has been shown that the STAT5 protein level is modulated by both miRs in different cellular contexts and that STAT5 is a direct miR-222 and miR-223 target [18–20]. This highlighted a role that miR-222, miR-223 or both may play in the post-transcriptional regulation of STAT5A upon EV treatment. Indeed, although both miRs increased in MCs that had been subjected to EV challenge, only miR-222 over-expression seems to be relevant in driving EV biological response in MCs. As a matter of fact, over-expressing miR-222, but not miR-223 led to a STAT5A level that was almost undetectable, miR-21 that was down-regulated as well as TGFβ expression and collagen production that were barely discernible. Moreover, experiments into transcription blockades by alpha-amanitin suggested that miR-222 expression in MCs relied on the transfer of this specific miR from EVs into recipient cells. Of particular interest is the fact that miR-222 over-expression did not have an effect on LG-cultured MCs. These results are consistent with the notion that miR(s) transferred into a recipient cell may drive discrete biological programs [48], depending on the target cell and specific biological context. EVs have also been shown to display a therapeutic effect via the transfer of various miRs with protective activity [22,23,27,30]. Other potential therapeutic miRs were identified by investigating MSC and HLSC EV content, including miR-100 [27], which has been shown to target mTOR [37]. Although miR-100 content increases in HG-treated MC cells, possibly as a protective mechanism, its expression did not change upon EV treatment. However, as the balance of intracellular miR content that occurs after EV treatment is able to change the biological response of the cell, we attempted to mimic such an event by performing gain-of-function experiments using pre-miR-100. We herein provide evidence that the increase in miR-100 content in HG-treated cells is associated with the reduced expression of mTOR, TGFβ and collagen production. Although the biological effects exerted by EV administration are under extensive investigations the mechanisms behind miRs and proteins crosstalk inside the target cell are not completely understood. It is well established that EVs may modulate target cell functions by delivering a variety of miRs which alone or in combination could change recipient cell epigenome [24]. This is particularly true for miR-222 which post-transcripionally regulates STAT5 expression and indirectly *miPPR21* gene expression and miR-21 cellular content, via STAT5 transcriptional activity. This, in turn, by changing the balance between miR-21 and miR-100, may result in miR-100 post-transcriptional regulation of mTOR. We did not provide data on a direct correlation between miR-21 and miR-100. However, the results of the present study suggest that EVs, by transferring their cargo, might exert a fine tuning effect on endogenous miRs which may eventually translate in additional healing properties.

Different mechanisms can account for the failure of EVs to influence LG-MC functional behaviour. Differences in gene expression are expected in MCs subjected to LG and HG. Therefore, it is tempting to hypothesize that EV treatment may virtually inhibit HG-mediated gene expression via mechanisms including RNA interference, giving EVs a precise therapeutic potential. This may confer to EVs additional therapeutic benefits to EVs by overcoming the major safety concerns.

Mitochondrial dysfunction plays a crucial role in HG-mediated damage [38]. We herein report that HG treatment is associated with the down-regulation of a nuclear encoded mitochondrial ETC component, CoxIV. It has been suggested that the expression of ETC component(s) could compensate harmful cues [38]. It is worth noting that EV administration boosted CoxIV expression, indicating that the fine-tuning of CoxIV can be driven by EV-mediated miR-21 down-regulation. Indeed, miR-21 is known to be involved in mitochondrial functional activity [39]. However, our results on LG-cultured MCs that over-express miR-21 and HG-cultured cells depleted of miR-21 indicate that changes in the intracellular miR-21 content cannot account for the deranged mitochondrial function in the hyperglycaemic setting on their own. Nevertheless, the results presented herein suggest that EVs can also protect MCs from harmful mitochondrial signals by transferring their cargo in a hyperglycaemic setting.

**Fig 7:**
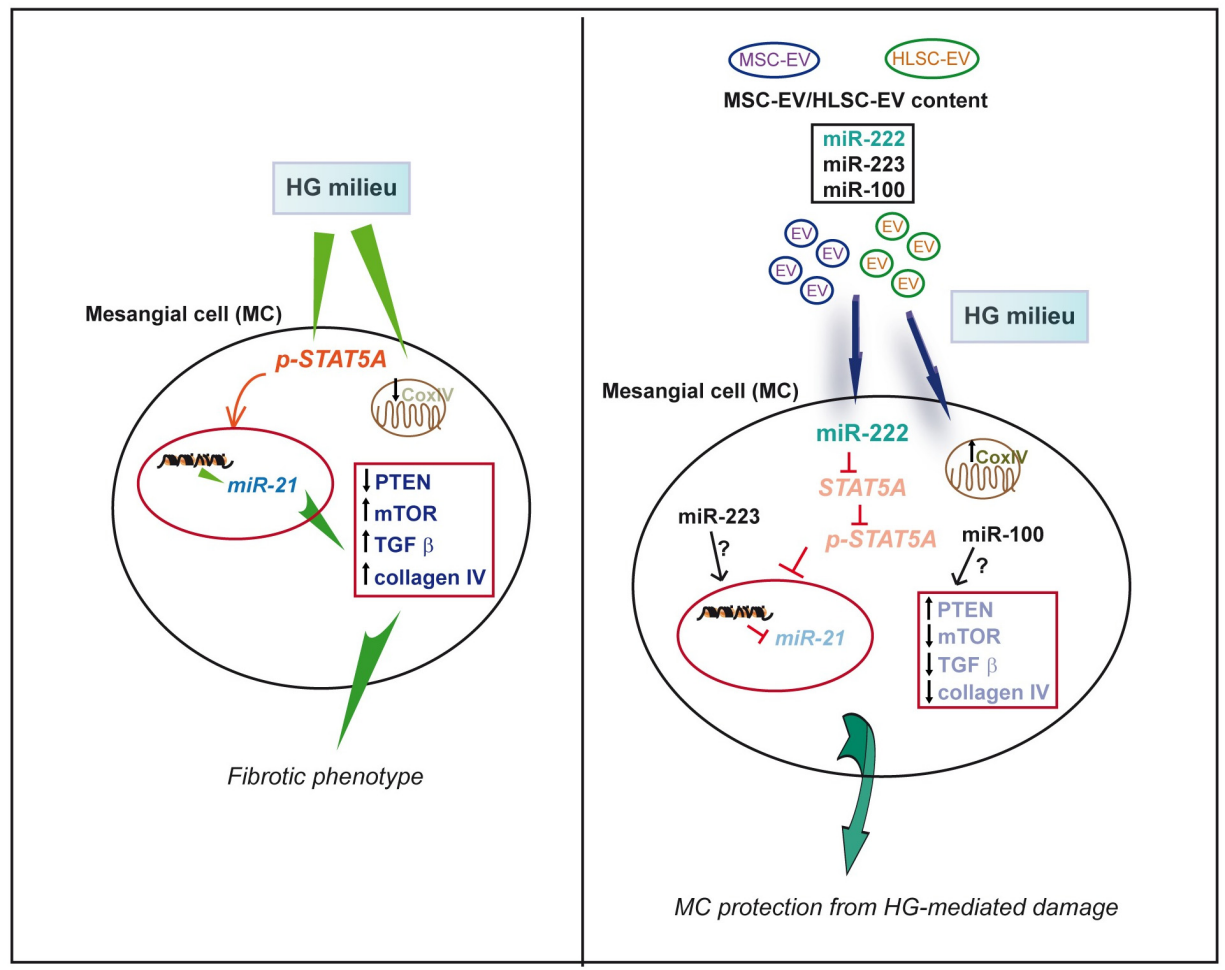
Schematic representation of the mechanisms through which MSC/HLSC microRNA-carrying vesicles protect MCs from collagen production in hyperglycaemic setting. Hyperglycaemic conditions lead to matrix production via STAT5A and miR-21 up-regulation. This translates into PTEN/mTOR and TGFβ expression and collagen type IV production. MC treated with MSC- and HLSC-derived EVs are protected from fibrogenic signals via the release of EV cargo; miR-222 post-transcriptionally regulates STAT5A which in turn controls miR-21 expression. Changes in the balance between miR-21 and miR-100 may also contribute to EV action. Finally, MSC- and HLSC-EV treatment also saves MCs from harmful mitochondrial signals by up-regulating CoxIV expression.

EVs are recognized as an integral component of the cell-to-cell communication network involved in tissue regeneration [49] and may actively transfer various molecules to target cells. The consequent modulation of cell phenotype is mainly due to transcriptional and stable epigenetic changes [50]. Indeed, we herein demonstrate that EVs released from MSCs and HLSCs display therapeutic potential in protecting MCs from HG-mediated collagen production. This occurs via the horizontal transfer of functional miR-222, and direct interference with damaging cues (Fig 7). Moreover, the results of the present study indicate that the release of EV cargo into the recipient cell may provide additional therapeutic advantages, including interference with mitochondrial dysfunction, by altering the balance of endogenous miR(s).

## Supporting Information

S1 Fig Gain- and loss-of-function experiments. (A-B) Gain-of-function experiments were performed on MCs that had either been cultured in LG or HG medium for 48h, using pre-miR-222 (A) or premiR-223 (B) oligonucleotides. Pre-miR negative control (neg c) oligonucleotides were used as a control. miR-222 and miR-223 expression was evaluated by qRT-PCR and normalized to RNU6B (five experiment performed in triplicate, n = 5) (p = 0.04, pre-miR-222 vs pre miR neg c in LG; p = 0.001, pre-miR-222 vs pre miR neg in HG in A; p = 0.012, pre-miR-223 vs pre miR neg c in LG; p = 0.004, pre-miR-223 vs pre miR neg in HG in B). (C) MCs incubated in the presence of 50 μg/ml of α-amanitin to inhibit MC transcription were either stimulated with MSC-EVs, HLSC-EVs or not at, all as well as being pre-treated with RNAse or left untreated. EV-miR-222 transfer was evaluated by q-RT-PCR. The difference in Ct values (ΔCt) between α-amanitin-treated MCs alone or those with the indicated EVs is reported (p<0.001) (mean±SEM). (D-E) miR-21 expression was evaluated using qRT-PCR and normalized to RNU6B (five experiments performed in triplicate, n = 5) on LG-cultured MCs transfected with either pre-miR neg c or pre-miR-21, and on HGcultured MCs transfected with either anti-miR negative control or anti-miR-21 (p = 0.03, pre-miR-21 vs pre miR neg c in LG in D; p = 0.02, anti-miR-21 vs anti-miR neg in HG in E).
(PDF)

S1 Table Reagents and antibodies. (DOCX)
