# Supplementary material for: Stem Cell-Derived, microRNA-Carrying Extracellular Vesicles: A Novel Approach to Interfering with Mesangial Cell Collagen Production in a Hyperglycaemic Setting
Source: PLoS One. 2016 Sep 9;11(9):e0162417. doi: 10.1371/journal.pone.0162417 (PMC5017750; doi:10.1371/journal.pone.0162417)
Supplement: S1 Fig — (A-B) Gain-of-function experiments were performed on MCs that had either been cultured in LG or HG medium for 48h, using pre-miR-222 (A) or premiR-223 (B) oligonucleotides. Pre-miR negative control (neg c) oligonucleotides were used as a control. miR-222 and miR-223 expression was evaluated by qRT-PCR and normalized to RNU6B (five experiment performed in triplicate, n = 5) (p = 0.04, pre-miR-222 vs pre miR neg c in LG; p = 0.001, pre-miR-222 vs pre miR neg in HG in A; p = 0.012, pre-miR-223 vs pre miR neg c in LG; p = 0.004, pre-miR-223 vs pre miR neg in HG in B). (C) MCs incubated in the presence of 50 μg/ml of α-amanitin to inhibit MC transcription were either stimulated with MSC-EVs, HLSC-EVs or not at, all as well as being pre-treated with RNAse or left untreated. EV-miR-222 transfer was evaluated by q-RT-PCR. The difference in Ct values (ΔCt) between α-amanitin-treated MCs alone or those with the indicated EVs is reported (p<0.001) (mean±SEM). (D-E) miR-21 expression was evaluated using qRT-PCR and normalized to RNU6B (five experiments performed in triplicate, n = 5) on LG-cultured MCs transfected with either pre-miR neg c or pre-miR-21, and on HGcultured MCs transfected with either anti-miR negative control or anti-miR-21 (p = 0.03, pre-miR-21 vs pre miR neg c in LG in D; p = 0.02, anti-miR-21 vs anti-miR neg in HG in E). (PDF) [file pone.0162417.s001.pdf]

S1 Fig

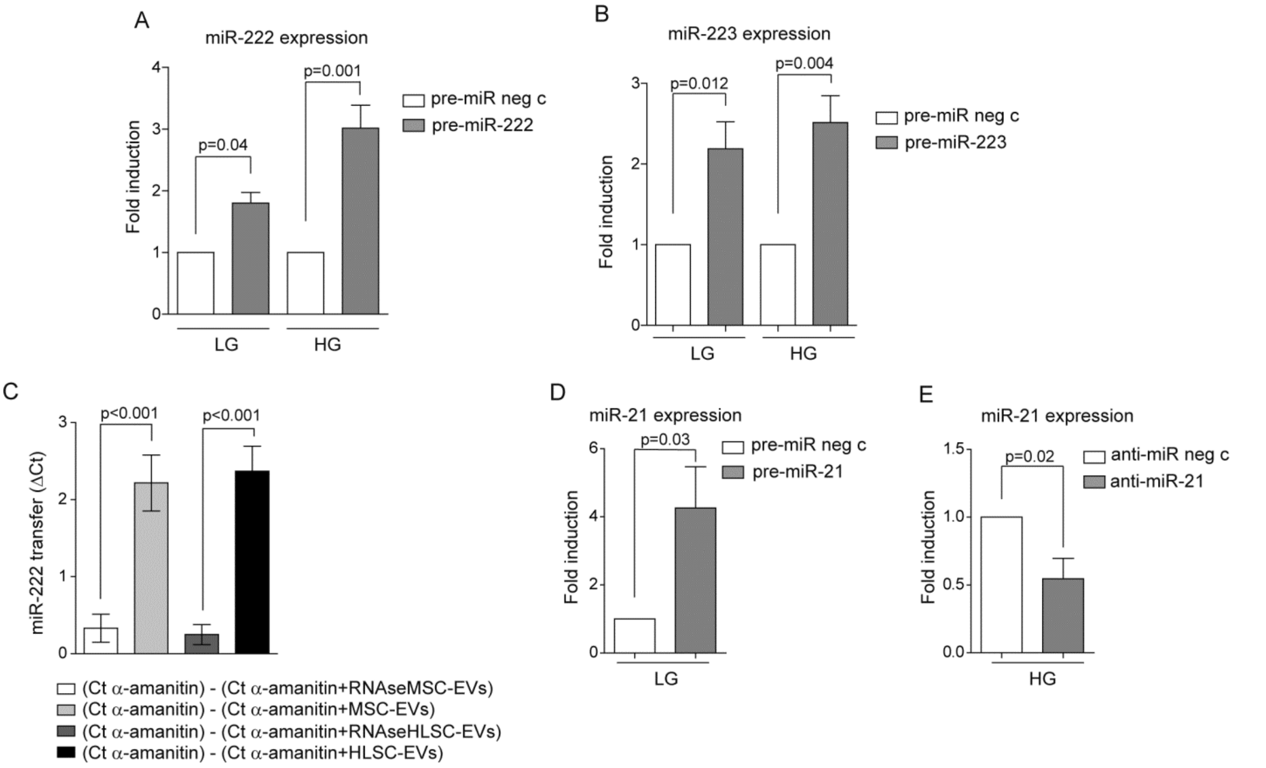

Gain- and loss-of-function experiments. (A-B) Gain-of-function experiments were performed on MCs that had either been cultured in LG or HG medium for 48h, using pre-miR-222 **(A)** or pre-miR-223 **(B)** oligonucleotides. Pre-miR negative control (neg c) oligonucleotides were used as a control. miR-222 and miR-223 expression was evaluated by qRT-PCR and normalized to RNU6B (five experiment performed in triplicate, n=5) (*p=0.04*, pre-miR-222 vs pre miR neg c in LG; *p=0.001*, pre-miR-222 vs pre miR neg in HG in **A**; *p=0.012*, pre-miR-223 vs pre miR neg c in LG; *p=0.004*, pre-miR-223 vs pre miR neg in HG in **B**). **(C)** MCs incubated in the presence of 50 µg/ml of α-amanitin to inhibit MC transcription were either stimulated with MSC-EVs, HLSC-EVs or not at, all as well as being pre-treated with RNAse or left untreated. EV-miR-222 transfer was evaluated by q-RT-PCR. The difference in Ct values (ΔCt) between α-amanitin-treated MCs alone or those with the indicated EVs is reported (*p<0.001*) (mean±SEM). (**D-E)** miR-21 expression was evaluated using qRT-PCR and normalized to RNU6B (five experiments performed in triplicate, n=5) on LG-cultured MCs transfected with either pre-miR neg c or pre-miR-21, and on HG-cultured MCs transfected with either anti-miR negative control or anti-miR-21 (*p=0.03*, pre-miR-21 vs pre miR neg c in LG in **D**; *p=0.02*, anti-miR-21 vs anti-miR neg in HG in **E**).
