# Supplementary material for: Stem Cell-Derived, microRNA-Carrying Extracellular Vesicles: A Novel Approach to Interfering with Mesangial Cell Collagen Production in a Hyperglycaemic Setting
Source: PLoS One. 2016 Sep 9;11(9):e0162417. doi: 10.1371/journal.pone.0162417 (PMC5017750; doi:10.1371/journal.pone.0162417)
Supplement: S1 Table — (DOCX) [file pone.0162417.s002.docx]

**S1 Table.**

**Reagents and antibodies**

| **Reagents** | | |
| --- | --- | --- |
| **Description:** | | **Purchased from:** |
| Bovine calf serum (BSA) (A8806), FCS (12138C), FBS (F6178), RNase (R6513), SDS (L3771), PIPES (P9291), Triton X-100 (T8787), Nonidet P-40 (74385), NaCl (S3014), NaF (S7920), Na_3_OV_4_ (S6508), Na_4_P_2_O_7_ (P8010), MgCl_2_ (M8266), KCl (P9541), HCl (258148), Na-azide (S2002), Hepes (H3375), Tris (T1503), EDTA (E6758), EGTA (E4378), ethanol (51976), aprotinin (A6279), pepstatin A (P5318), PMSF (P7626), DMSO (D8418), DMEM 5 mmol/l (D5546), DMEM 25 mmol/l (D5671), leupeptin (L2884), penicillin-streptomycin (P4333), Trypsin (T4799), α-amanitin (A2263), RIPA buffer (R0278) | | Sigma-Aldrich (St Louis, MO, USA) |
| Protein molecular weight markers (161-0374), Acrylammide (161-0156), polyvinylidene difluoride (PVDF) membranes (162-0115), Bradford reagent (500-0205), ECL (170-5061) | | Bio-Rad (Hercules, CA, USA) |
| Lipofectin® Reagent (18292-037), TRIZOL (15596018),  hsa-miR-222 (002276), hsa-miR-223 (002295), RNU6B (001093) pre-miR-21 (PM10206), pre-miR-222, (PM11376) pre-miR-223 (PM12301), pre-miR-100-5p (PM10188), anti-miR-21-5p (AM10206) | | Invitrogen^TM^ (Life Technologies Carlsbad, CA, USA; Paisley, UK). |
| Syber Green primers:  -RNU6B: *CGCAAGGATGACACGCAA*  -miR-21-5p (MIMAT0000076): *TAGCTTATCAGACTGATGTTGA*  -miR-100-5p (MIMA0000098): *AACCCGTAGATCCGAAGTTGTG* | | Quiagen (Valencia, CA, USA) |
| EndoGRO Medium (SCME-004) | | Merck Spa (Milan, Italy) |
| Dual Luciferase reporter Assay System (E1910) | | Promega Corporation (Madison, WI, USA) |
| **Antibodies** | | |
| **Description:** | **Purchased from:** | |
| anti-β actin (sc-47778)  anti-collagen type IV (sc-70246)  anti-STAT5A (sc-1081) | S. Cruz Biotechnology (Heidelberg, Germany) | |
| anti-TGFβ (ab9758)  anti-CoxIV (ab14744) | Abcam (Cambridge, UK) | |
| anti-rabbit IgG, HRP linked (4050-05)  anti-mouse IgG, HRP linked (1031-05) | Southern Biotech (Birmingham, Alabama USA) | |
| anti-pSTAT5 (9314S)  anti-PTEN (9552S) | Cell Signaling Technology (Danvers MA, USA) | |
